# Supplementary material for: Demand–supply-side barriers affecting maternal health service utilization among rural women of West Shoa Zone, Oromia, Ethiopia: A qualitative study
Source: PLoS One. 2022 Sep 28;17(9):e0274018. doi: 10.1371/journal.pone.0274018 (PMC9518902; doi:10.1371/journal.pone.0274018)
Supplement: S1 File — (DOCX) [file pone.0274018.s001.docx]

# Annex

**Data collection tools**

**Key Informant Interview Guide**

1. Position of the interviewed person_________________________________
2. General community information (No. of HEWS, health posts, HDA and their functionality).
3. What is the average distance of the communities from their nearest catchment health center within your sub-district to the facility?
4. What maternal and newborn intervention programmes do you run in the catchment area of your facility?
5. Community demand and preconditions for services, the overall service status.
6. What challenges does the facility experience in executing health education relating to maternal and new-born health?
7. Community experiences utilization and reason for not utilizing (ANC, SBA, PNC)
8. What institutional challenges are you facing in carrying out maternal health programmes?
9. To what extent do community leaders assist during any intervention programmes implemented by the facility?
10. What suggestions can you make to help improve maternal health care utalization in this district?
11. Community experience with services and perceived quality of the services
12. Reporting system (incompleteness, false, language difficulties).
13. Observational check lists for auditing availability of the equipments needed for service.
14. Are there any local initiatives by the community to prevent maternal, If yes, please give details of those programs.

**Health Extension Workers Interview Guide**

1. Introduce yourself and your roles.
2. Roles and responsibility of HEWs?
3. How do you see the maternal health service delivery: ANC, SBA, and PNC in your kebele? (MERITS AND CHALLENGES HERE)
4. What barriers do you think is affecting the MH service utilization(from HI vs community side, women side).
5. What arrangements are put in place for ANC, PNC and emergency referrals?
6. What is you perception on why some pregnant mother refused to use antenatal care services or complete the recommended number of visits? Or SBA and PNC?
7. What is your broad view about the challenges HEWs face in carrying out duty of care to clients (antenatal care, women in labor or postnatal care)?
8. In your opinion, what recommendations will you make for improved maternal health outcomes in the rural/hard-to-reach areas?
9. Over all, what recommendation/suggestion you have about general MH service issue?

**Focal Group Discussion guidelines for Women participants**

1. What services do you know the services that a mother should get during pregnancy, delivery and after delivery?
2. How do you express the service utilizing schedule? For example ANC? How many times, where, when?
3. What are the benefits of ANC?
4. Did you used or the women in your area are utilizing this service sufficiently? If yes, how? If no why?
5. Family (husband, mother in law) and community support for ANC?
6. Where the majority of the women in your community are giving birth? Including your experience?
7. What do you think the reason for not utilizing delivery at health institution?
8. What do you think should be done for women after delivery, what service should she get?
9. What do you think should be done to overcome these problems?
10. Have you being visited by health professional or HEWs at your home in the first 42 days after you gave births?
11. How do you explain support from your family i.e. husband or mother in-law or father in law to utilize MH services?
12. How do you perceive about the MH services, counseling and ANC, being delivered in your kebele by HEWs? Good or poor? Why?
13. Over all, what do you think should be done to increase service utilization in your area? Who should do what?
