# Supplementary material for: Demand–supply-side barriers affecting maternal health service utilization among rural women of West Shoa Zone, Oromia, Ethiopia: A qualitative study
Source: PLoS One. 2022 Sep 28;17(9):e0274018. doi: 10.1371/journal.pone.0274018 (PMC9518902; doi:10.1371/journal.pone.0274018)
Supplement: S2 File — (DOC) [file pone.0274018.s002.doc]

**Consolidated criteria for reporting qualitative studies (COREQ): 32-item checklist**

**Manuscript title:** ***Demand –supply-side barriers affecting maternal health service utilization among rural women of Ethiopia: A qualitative study***

| **No. Item** | **Guide questions/description** | **Reported on Page #** |
| --- | --- | --- |
| **Domain 1: Research team and reﬂexivity** |  |  |
| *Personal Characteristics* |  |  |
| 1. Inter viewer/facilitator | Which author/s conducted the inter view or focus group? | Page 6 |
| 2. Credentials | What were the researcher’s credentials? E.g. PhD, MD | Page 1 |
| 3. Occupation | What was their occupation at the time of the study? | Page 1 and 6 |
| 4. Gender | Was the researcher male or female? | Both |
| 5. Experience and training | What experience or training did the researcher have? | All researchers have both training and previous Qualitative research experience. |
| *Relationship with participants* |  |  |
| 6. Relationship established | Was a relationship established prior to study commencement? | Yes. Page 5 and 6  (under subsection b & d)  . |
| 7. Participant knowledge of the interviewer | What did the participants know about the researcher? e.g. personal goals, reasons for doing the research | The overall purpose of the study was clearly briefed for the participants.  Page 7, under subsection Ethical issue |
| 8. Interviewer characteristics | What characteristics were reported about the inter viewer/facilitator? e.g. Bias, assumptions, reasons and interests in the research topic | The FGD facilitators has no interest with the issue being studied. They were recruited only for the data collection purpose. PIs were engaged as Supervisors to ensure the quality of collected data |

| **Domain 2: study design** |  |  |
| --- | --- | --- |
| *Theoretical framework* |  |  |
| 9. Methodological orientation and Theory | What methodological orientation was stated to underpin the study? e.g. grounded theory, discourse analysis, ethnography, phenomenology, content analysis | Page 5 and 6 |
| *Participant selection* |  |  |
| 10. Sampling | How were participants selected? e.g. purposive, convenience, consecutive, snowball | Page 5 underSubsection: Study Population and sampling techniques |
| 11. Method of approach | How were participants approached? e.g. face-to-face, telephone, mail, email | Face to face  Page-6 |
| 12. Sample size | How many participants were in the study? | Page 5 and 7 |
| 13. Non-participation | How many people refused to participate or dropped out? Reasons? | None |
| *Setting* |  |  |
| 14. Setting of data collection | Where was the data collected? e.g. home, clinic, workplace | At health post and at office  Page: 5  . |
| 15. Presence of non-participants | Was anyone else present besides the participants and researchers? | No |
| 16. Description of sample | What are the important characteristics of the sample? e.g. demographic data, date | Page 7  under Result part |
| *Data collection* |  |  |
| 17. Interview guide | Were questions, prompts, guides provided by the authors? Was it pilot tested? | Additional file attached and page 6 |
| 18. Repeat interviews | Were repeat inter views carried out? If yes, how many? | No |
| 19. Audio/visual recording | Did the research use audio or visual recording to collect the data? | Audio page: |
| 20. Field notes | Were ﬁeld notes made during and/or after the inter view or focus group? | Yes, Page 6 |
| 21. Duration | What was the duration of the inter views or focus group? | Page 5 |
| 22. Data saturation | Was data saturation discussed? | Page 5. |
| 23. Transcripts returned | Were transcripts returned to participants for comment and/or correction? | No |
| **Domain 3: analysis and ﬁndings** | | |
| *Data analysis* |  |  |
| 24. Number of data coders | How many data coders coded the data? | (Two experts who have an experience with MAXQDA software)  Page 6 |
| 25. Description of the coding tree | Did authors provide a description of the coding tree? | Yes page 8 (Table 1) and page 16 (Fig:1) |
| 26. Derivation of themes | Were themes identiﬁed in advance or derived from the data? | Page 7 and 8 |
| 27. Software | What software, if applicable, was used to manage the data? | MAXQDA –software  Page-6 |
| 28. Participant checking | Did participants provide feedback on the ﬁndings? | Not yet. We are trying |
| *Reporting* |  |  |
| 29. Quotations presented | Were participant quotations presented to illustrate the themes/ﬁndings? Was each quotation identiﬁed? e.g. participant number | Yes:  The whole Result part. |
| 30. Data and ﬁndings consistent | Was there consistency between the data presented and the ﬁndings? | Yes, |
| 31. Clarity of major themes | Were major themes clearly presented in the ﬁndings? | Yes.  Table 1 on page 8 |
| 32. Clarity of minor themes | Is there a description of diverse cases or discussion of minor themes? | Discussion of major and minor themes on page 7 and 8 |
