# Supplementary figures and images for: Demand–supply-side barriers affecting maternal health service utilization among rural women of West Shoa Zone, Oromia, Ethiopia: A qualitative study
Source: PLoS One. 2022 Sep 28;17(9):e0274018. doi: 10.1371/journal.pone.0274018 (PMC9518902; doi:10.1371/journal.pone.0274018)

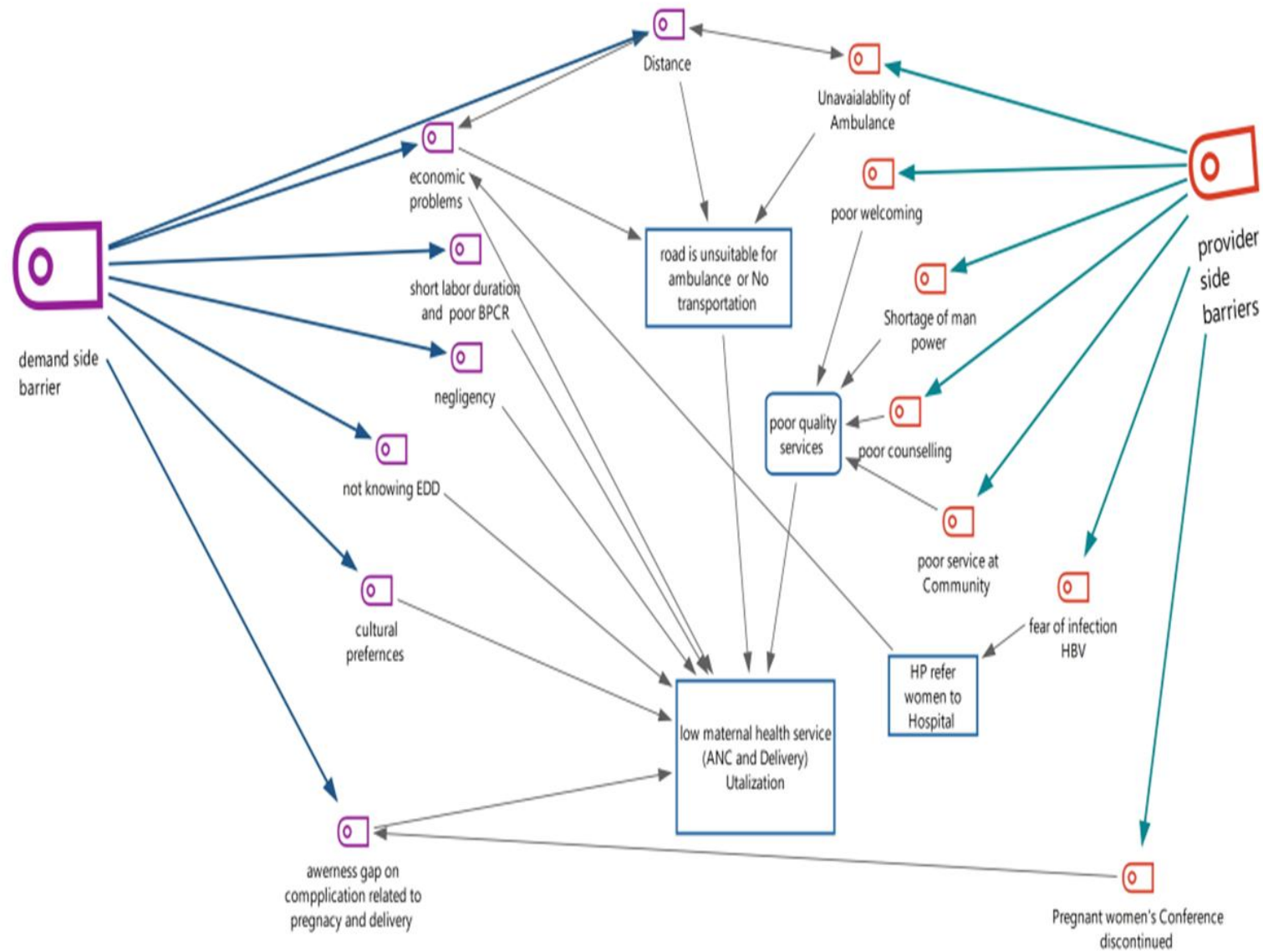

Fig.1 Indicating Barriers to maternal Health Service Utilization and their interactions...

Supplement: S1 Fig — (PDF) [file pone.0274018.s003.pdf]
